# Exploration of changes in the brain response to sleep-related pictures after cognitive–behavioral therapy for psychophysiological insomnia

**DOI:** 10.1038/s41598-017-13065-0

**Published:** 2017-10-02

**Authors:** Seog Ju Kim, Yu Jin Lee, Nambeom Kim, Soohyun Kim, Jae-Won Choi, Juhyun Park, Ah Reum Gwak, Chang-Ki Kang, Seung-Gul Kang, Do-Un Jeong

**Affiliations:** 1 Department of Psychiatry, Sungkyunkwan University College of Medicine, Samsung Medical Center, Seoul, Republic of Korea; 2 Department of Psychiatry and Center for Sleep and Chronobiology, Seoul National University College of Medicine and Hospital, Seoul, Republic of Korea; 3 Neuroscience Research Institute, Gachon University, Incheon, Republic of Korea; 4 Department of Psychology, University at Buffalo, New York, USA; 5 Department of Radiological Science, Gachon University, Incheon, Republic of Korea; 6 Department of Psychiatry, Gil Medical Center, School of Medicine, Gachon University, Incheon, Korea

## Abstract

Psychophysiological insomnia (PI) includes arousal to sleep-related stimuli (SS), which can be treated by cognitive behavioral therapy for insomnia (CBT-I). The present study was an exploratory, prospective intervention study that aimed to explore brain response to visual SS in PI before and after CBT-I. Blood oxygen level dependent (BOLD) signal differences in response to SS and neutral stimuli (NS) were compared between 14 drug-free PI patients and 18 good sleepers (GS) using functional magnetic resonance imaging (fMRI). BOLD changes after CBT-I in patients were also examined. PI patients showed higher BOLD activation to SS in the precentral, prefrontal, fusiform, and posterior cingulate cortices before CBT-I. The increased responses to SS were reduced after CBT-I. The increased response to SS in the precentral cortex was associated with longer wake time after sleep onset (WASO), and its reduction after CBT-I was associated with improvements in WASO. Clinical improvements after CBT-I were correlated with BOLD reduction in the right insula and left paracentral cortex in response to SS. PI showed hyper-responses to SS in the precentral cortex, prefrontal cortex, and default mode network and these brain hyper-responses were normalized after CBT-I. CBT-I may exert its treatment effects on PI by reducing hyper-responses to SS in the precentral cortex and insula.

## Introduction

Insomnia symptoms can be accompanied by various psychiatric and medical illnesses. However, the International Classification of Sleep Disorders (ICSD) regards insomnia disorder as an independent illness rather than as merely dependent on other conditions^1^. The diagnosis of insomnia disorder can be made when sleep difficulties and related daytime dysfunctions are present despite adequate time and opportunity for sleep.

Psychophysiological insomnia (PI), one such example of an independent insomnia that has its own pathophysiology, is known to be a learned insomnia, caused by a conditioning process whereby sleep-related conditions and hyperarousal are paired. Poor sleep in PI increases sleep-related anxiety, which further exacerbates hyperarousal. Through repeated experiences of sleepless nights, patients with PI become more preoccupied with ‘good’ sleep^2^.

The attention-intention-effort (AIE) pathway model has been proposed as a cognitive model for PI^2^. The AIE pathway model argues that PI develops and is maintained by three processes: 1) attention to sleep-related stimuli (SS), 2) intention to sleep, and 3) effort to sleep. Excessive attention, intent, and effort to achieve good sleep increase sleep-related anxiety, thus maintaining insomnia. Another cognitive model of insomnia^3^ suggests that excessive worry and fear over disrupted sleep renders individuals anxious and aroused. For both models, selective or exaggerated attention to SS can be produced in PI patients due to excessive preoccupation with sleep.

Previous neurophysiological research has shown that patients with insomnia usually show hyperarousal in response to SS. Insomnia patients pay more attention^4–6^ and show elevated cognitive and psychological arousal to SS^7^. In a recent review^8^, the majority of research comparing good sleepers and insomnia patients reported sleep-related attentional bias in insomnia patients. However, only two functional magnetic resonance imaging (fMRI) studies of insomnia investigating brain activation in response to SS have been conducted by a single research group^9,10^. These authors showed increased amygdala activity in insomnia patients in response to insomnia-related pictures, but they were unable to find any differences in brain activity between insomnia patients and good sleepers (GS) in response to sleep-related words.

The treatment of choice for PI is cognitive behavioral therapy for insomnia (CBT-I)^11–13^. CBT-I is intended to correct the psychophysiological component of the insomnia. CBT-I techniques try to decrease the potential conditioned pairing between awakening and sleep-related information^14^. Thus, after successful CBT-I, PI patients are expected not to experience hyperarousal in bed and not to overreact to SS.

Little is known about how CBT-I exerts its effects on sleep-related attentional bias in PI patients. Only one reported fMRI study has compared brain activation before and after CBT-I in insomnia patients^15^, showing prefrontal hypo-activation during a verbal fluency task that was recovered by CBT-I. However, brain activity during cognitive tasks may be more related to the outcome of CBT-I, whereas selective attention or hyperarousal from sleep-related information is one of the targets of CBT-I. However, to our knowledge, no previous report has compared brain responses to SS before and after CBT-I.

The current study aimed to explore the brain response in drug-free PI patients to visual SS before and after CBT-I using fMRI. Based on the cognitive theories of PI and CBT-I, we set two hypotheses, as follows. First, we hypothesized that, compared with good sleepers (GS), patients with PI would show more activation in response to SS in insomnia-related areas, especially in the frontal cortex and limbic structures, before CBT-I. Second, we hypothesized that this increased brain response of PI patients to SS in these areas before CBT-I would be reduced after CBT-I.

## Results

### Comparison of clinical characteristics between PI and GS

**Figure 1:**
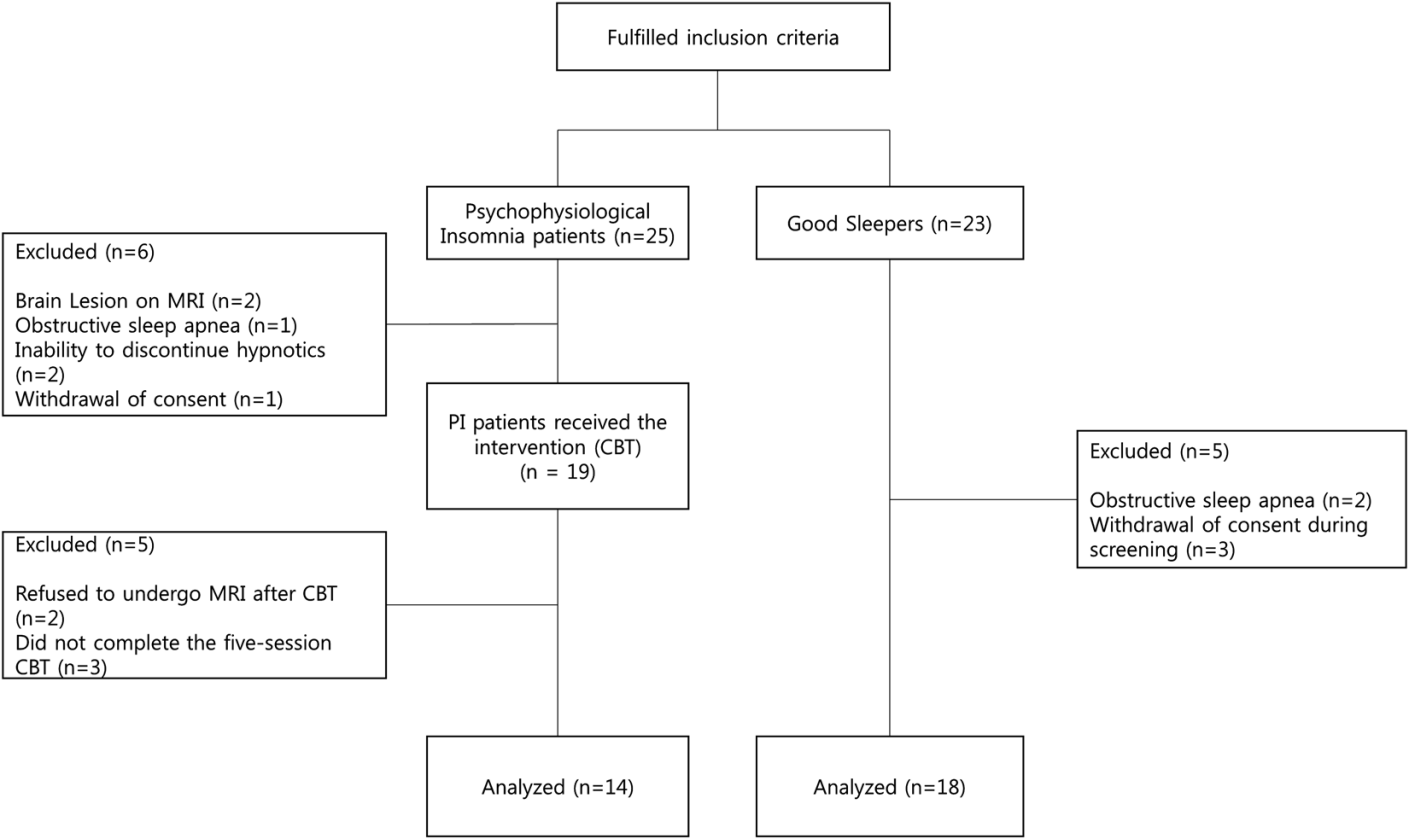
Diagram of the study flow of PI patients and GS. Abbreviations: PI: psychophysiological insomnia, GS: good sleepers.

The clinical characteristics of the study participants are presented in Table 1, and a diagram illustrating the flow of participants is presented in Fig. 1. There were no significant differences in age or gender between PI and GS. Compared with GS, PI patients showed higher scores on the Pittsburgh Sleep Quality Index (PSQI; t = 7.534, p < 0.001) and the Dysfunctional Beliefs and Attitudes about Sleep Scale-16 (DBAS-16; t = 4.290, p < 0.001). PSG showed that PI patients had lower total sleep time (TST; t = 2.541, p = 0.016) and higher WASO (t = 2.187, p = 0.037) than GS. PI patients also showed lower sleep efficiency (SE) than GS on PSG, although the difference was not significant (t = 2.030, p = 0.051).

**Table 1:** Comparison of clinical variables between the psychophysiological insomnia (PI) and good sleeper (GS) groups (df = 30).

	PI (*n* = 14)	GS (*n* = 18)	t	p-value
Age	49.0 ± 12.3	42.7 ± 12.3	1.443	0.159
Gender^a^	4 M, 10 F	4 M, 14 F	0.169	0.681
PSQI^b^**	13.4 ± 4.0	4.8 ± 2.5	7.534	<0.001
DBAS**	95.1 ± 19.8	59.7 ± 25.4	4.290	<0.001
BDI	9.5 ± 8.4	5.1 ± 5.4	1.800	0.082
**Nocturnal PSG**				
TIB (min)	475.5 ± 24.4	486.1 ± 11.9	−1.621	0.115
TST (min)*	411.0 ± 49.9	443.7 ± 19.1	−2.541	0.016
SE (%)	86.5 ± 9.1	91.3 ± 3.9	−2.030	0.051
SL (min)	11.0 ± 12.0	11.2 ± 10.2	−0.041	0.968
WASO (min)*	52.8 ± 38.0	30.9 ± 16.9	2.187	0.037
REML (min)	90.0 ± 39.4	92.6 ± 25.0	0.232	0.818
N1 (%)	13.1 ± 5.8	10.3 ± 5.5	1.375	0.179
N2 (%)	58.3 ± 9.1	61.0 ± 7.0	−0.951	0.349
N3 (%)	5.6 ± 5.0	6.5 ± 5.8	−0.492	0.626
REM (%)	23.1 ± 7.1	21.8 ± 3.8	0654	0.518

Note: Independent *t*-test, ^a^χ^2^ test, ^b^Scores ≥8.5 on the Korean version of the PSQI indicate poor sleep.

### Clinical change after CBT-I

Table 2 summarizes the clinical change between baseline, before the start of CBT-I, and after the completion of CBT-I in PI. After CBT-I, scores dropped significantly on the Insomnia Severity Index (ISI; t = 2.854, p = 0.014), PSQI (t = 5.559, p < 0.001), DBAS (t = 6.69, p < 0.001), and Beck Depression Inventory (BDI; t = 2.177, p = 0.049). In the sleep diaries, TST (t = 4.493, p = 0.001) and SE (t = 4.375, p = 0.001) increased significantly after CBT-I whereas sleep latency (t = 3.701, p = 0.003) and WASO (t = 2.720, p = 0.019) decreased significantly. A physician assessed each patient for any medical problems and their potential relationships with the CBT-I using open-ended questions in every CBT-I session. No specific adverse effects were plausibly induced by CBT-I, and no medical problems were reported by any PI patient during the CBT-I sessions.

**Table 2:** Changes in clinical variables before and after CBT-I in PI (*n* = 14).

	Before CBT-I Mean ± SD	After CBT-I Mean ± SD	t	p-value
ISI*	15.9 ± 9.0	7.0 ± 5.2	2.854	0.014
PSQI**	13.4 ± 4.0	6.9 ± 2.9	5.559	<0.001
DBAS**	95.1 ± 19.8	52.1 ± 32.4	6.690	<0.001
BDI*	9.5 ± 8.4	5.6 ± 7.8	2.177	0.049
**Sleep diary**				
TST (hr)**	5.5 ± 1.2	6.4 ± 0.8	−4.493	0.001
SL (min)**	39.5 ± 34.6	15.8 ± 15.8	3.701	0.003
WASO (min)*	65.2 ± 54.5	32.6 ± 32.7	2.720	0.019
SE (%)**	76.1 ± 14.1	88.9 ± 9.6	−4.375	0.001

### Comparison of brain activation in response to SS between PI and GS

**Figure 2:**
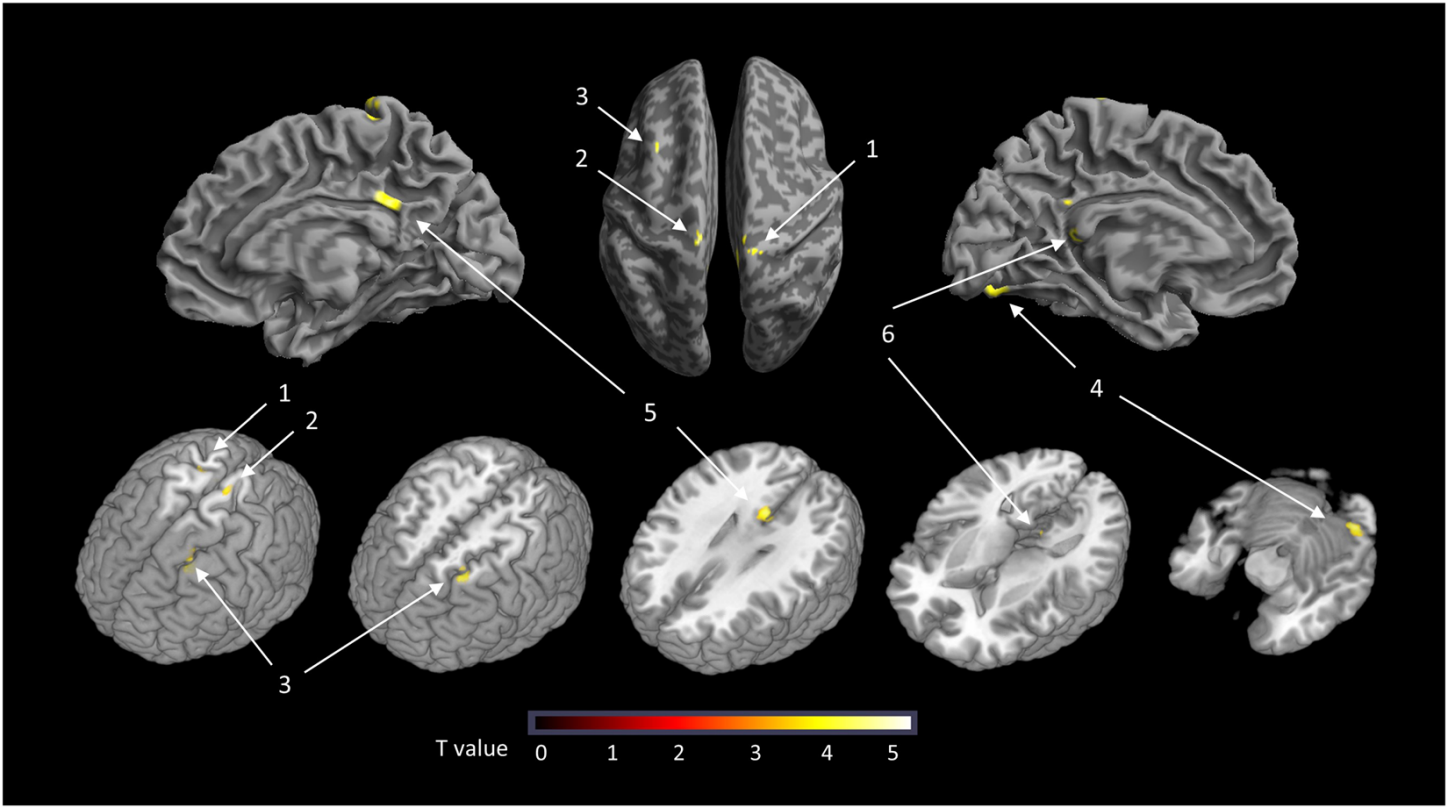
Brain areas showing a higher BOLD signal on response to SS in the PI versus GS group 1: Right precentral cortex 2: Left precentral cortex. 3: Left prefrontal cortex. 4: Left fusiform cortex. 5: Right posterior cingulate cortex. 6: Left posterior cingulate cortex. Note: BOLD response to SS was calculated by subtraction of the BOLD response to NS from that to SS (SS-NS).

BOLD signals to SS were significantly higher in PI than GS in the following brain areas (Table 3): the bilateral precentral cortex, left prefrontal cortex, left fusiform cortex, and bilateral posterior cingulate cortex (PCC; Fig. 2). When the five PI patients who did not complete the follow-up MRI scan were included in the analysis, all areas other than the left precentral cortex remained significant.

**Table 3:** Brain areas showing a higher BOLD signal in response to SS in the PI (n = 14) versus GS group (n = 18).

Regions	Brodmann Area	Cluster Size	Z value	Talairach coordinates		
				*x*	*y*	*z*
Right Precentral	4	49	3.69	12	−25	71
Left Precentral	4	36	3.7	−10	−23	69
Left Prefrontal	8	109	4.01	−26	25	50
Left Fusiform	18	90	4.48	−22	−78	−7
Right PCC	23	123	4.18	2	−37	31
Left PCC	29	23	3.62	−6	−38	11

Note: Z values refer to significant results.

According to these results, six regions of interest (ROIs) were defined as the voxel clusters showing significant between-group differences in BOLD signals in response to SS. The BOLD response to SS in the left precentral cortex of PI patients correlated positively with WASO in sleep diaries (r = 0.664, p = 0.020).

### Change in brain activation in response to SS after CBT-I

**Figure 3:**
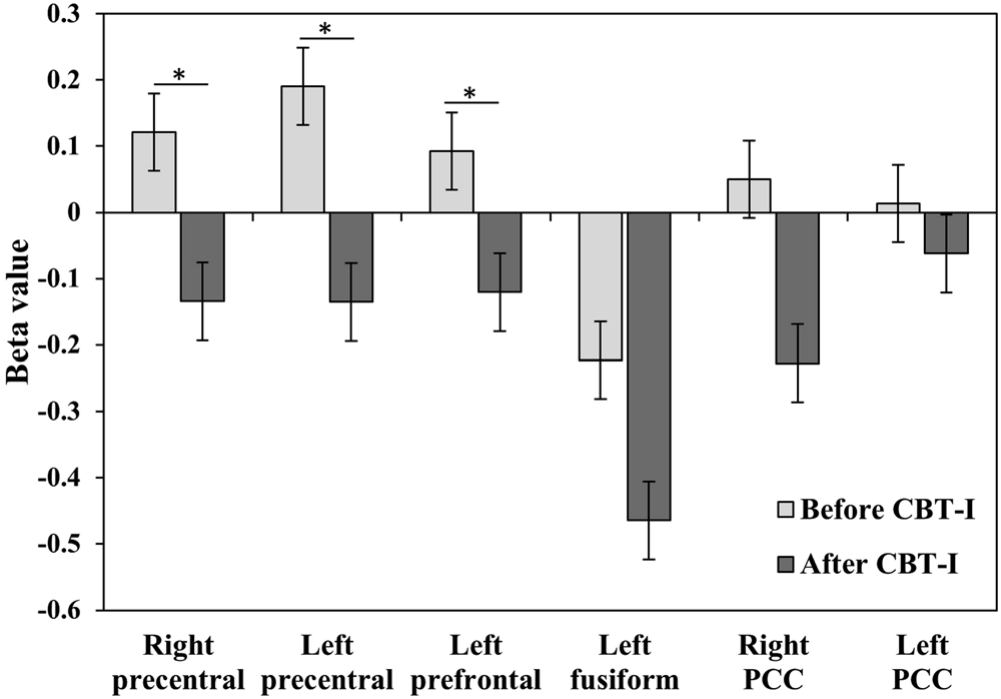
Regions of interest having higher BOLD signals in response to SS in PI, and changes in BOLD after CBT-I. Note: BOLD response to SS was calculated by subtraction of the BOLD response to NS from that to SS (SS-NS). Mean ± standard error.

BOLD responses to SS were reduced significantly in three of the pre-defined ROIs (Fig. 3): the right precentral cortex (t = 2.278, p = 0.040), the left precentral cortex (t = 2.322, p = 0.037), and the left prefrontal cortex (t = 2.615, p = 0.021). Although not statistically significant, the BOLD responses to SS were also decreased after CBT-I in the left fusiform cortex (t = 1.729, p = 0.107) and right PCC (t = 1.836, p = 0.089). Similar results were produced even after the exclusion of four PI subjects who were exposed to hypnotics and should have tapered their medications prior to the CBT-I. The BOLD responses to SS exhibited significant reductions in the right precentral cortex (t = 2.219, p = 0.042), left precentral cortex (t = 2.247, p = 0.024), and left prefrontal cortex (t = 2.779, p = 0.012) after excluding the hypnotics-exposed subjects. The reductions in BOLD responses to SS after CBT-I in the left precentral cortex correlated significantly with the increase in SE (r = −0.572, p = 0.041) and the decrease in WASO (r = 0.628, p = 0.022) after CBT-I (Supplementary Table 1 and Supplementary Fig. 1).

**Figure 4:**
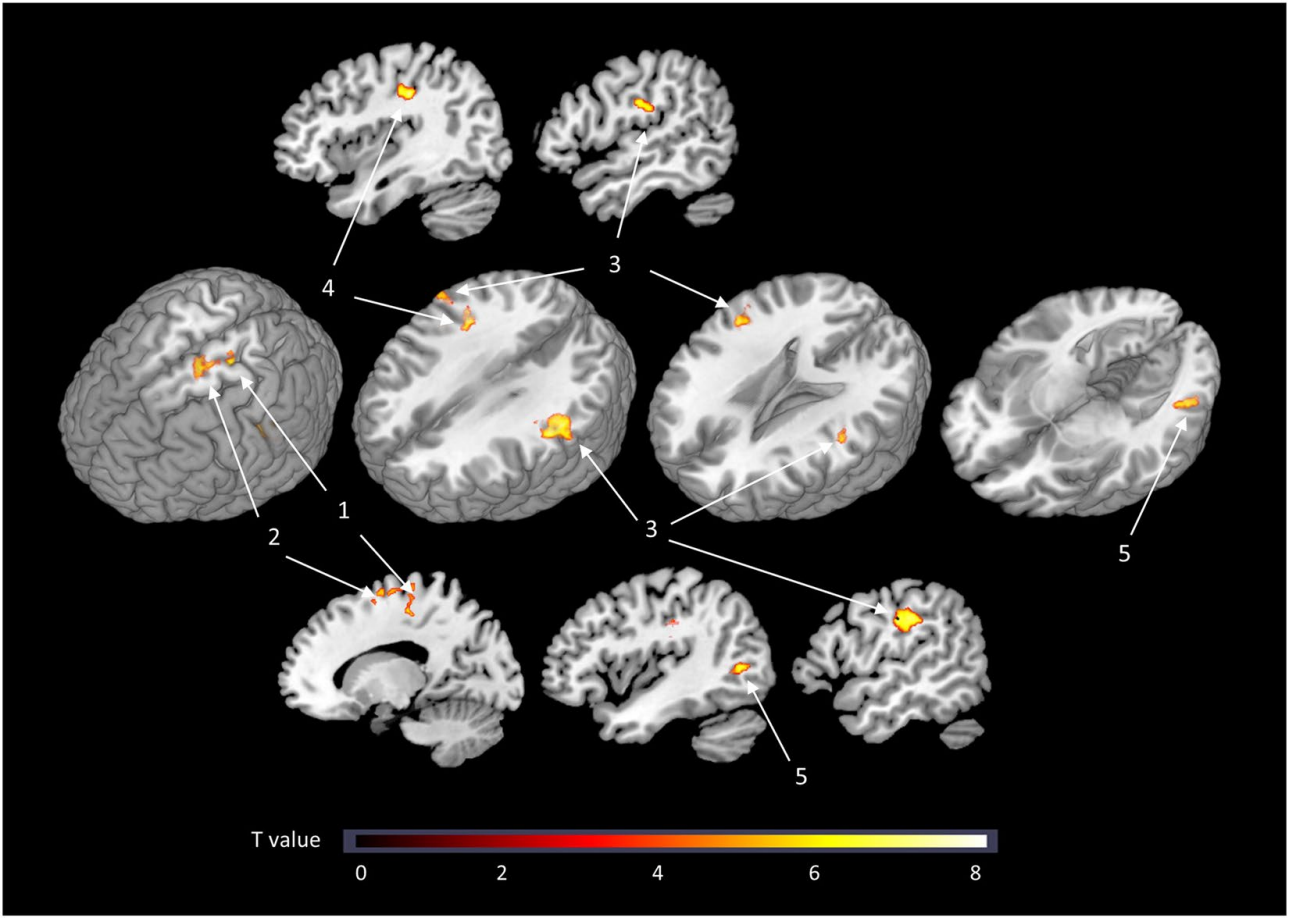
Brain areas showing a decreased BOLD response to SS after CBT-I in a voxel-wise whole-brain comparison. 1: Left precentral cortex. 2: Left supplementary motor cortex. 3: Bilateral supramarginal cortex. 4: Right postcentral cortex. 5: Left middle temporal cortex. Note: BOLD response to SS was calculated by subtraction of the BOLD response to NS from that to SS (SS - NS).

A voxel-wise whole-brain analysis was also conducted. The BOLD response to SS decreased after CBT-I in the following areas (Fig. 4): the left precentral cortex, right postcentral cortex, left supplementary motor cortex, bilateral supramarginal cortex, and left middle temporal cortex.

### Association between clinical effects and brain activation to SS after CBT-I

**Figure 5:**
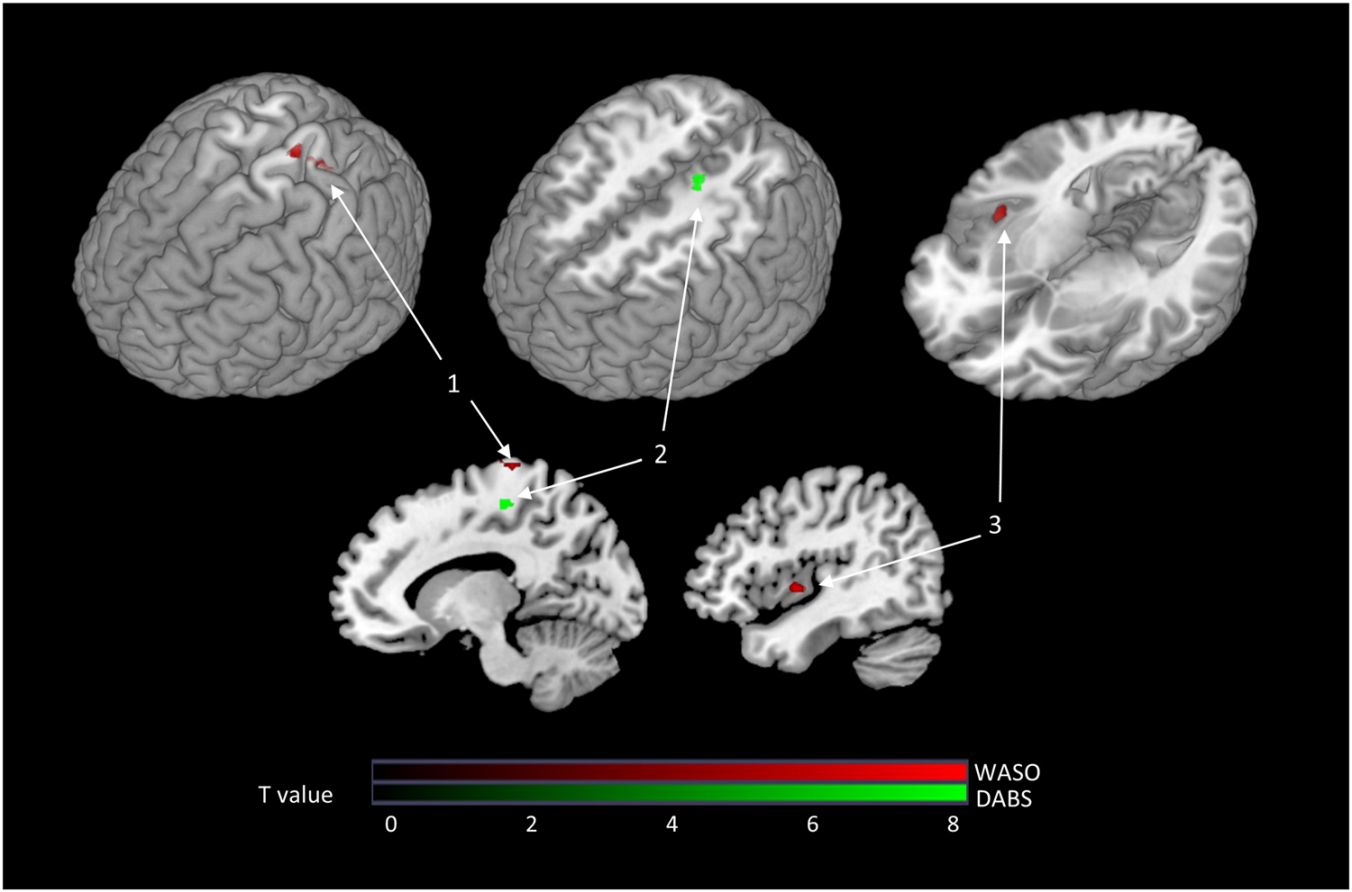
Brain areas where changes in the BOLD response to SS after CBT-I correlated significantly with clinical improvement in insomnia after CBT-I. 1: Left superior paracentral cortex. 2: Left inferior paracentral cortex. 3: Right insula. (**A**) Decrease in WASO after CBT-I was correlated with a reduction in the left superior paracentral BOLD response to SS. (**B**) DBAS score decrease after CBT-I correlated with a decrease in the left inferior paracentral BOLD response to SS. (**C**) WASO decrease after CBT-I correlated with a decrease in the right insular BOLD response to SS. Note: The BOLD response to SS was calculated by subtraction of the BOLD response to NS from the BOLD response to SS (SS-NS).

We also explored the whole-brain areas in which changes in the BOLD response to SS after CBT-I were correlated with clinical improvement using an SPM regression model (Fig. 5). The decrease in WASO following CBT-I was correlated with reductions in the BOLD response to SS in the right insula and left paracentral cortex after CBT-I. The decrease in DBAS after CBT-I was correlated with a reduced BOLD response to SS in the left paracentral cortex after CBT-I and with an enhanced BOLD response in the left parahippocampal and right middle frontal cortices.

## Discussion

In the current study, we showed that the precentral, prefrontal, and PCC areas of drug-free PI patients showed greater activation in response to SS. These increased responses to SS in the prefrontal and precentral cortices of drug-free PI patients were reduced after CBT-I. Additionally, clinical improvements after CBT-I (decreased WASO or increased SE) were correlated with a reduced response to SS in the precentral cortex and the insula after CBT-I. To our knowledge, this is the first report of changes in brain responses to SS after CBT-I.

Regarding our first hypothesis, we found increased activation to SS in several brain areas in patients with PI. This corresponds with the notion that brains in PI patients would be hyperactive to SS. Regarding our second hypothesis, CBT-I reduced this increased activation in response to SS in patients with PI, and these reductions correlated with the clinical efficacy of CBT-I, such as WASO or SE.

These findings suggest that there is a sleep-related attention bias in PI patients in terms of brain activity and indicate that the related brain hyper-activation may be normalized after CBT-I. Our findings also correspond to previous cognitive models of insomnia^2,3^. The AIE model posits that attention to SS, which occurs before intent or effort to sleep, plays an initial role in the development or maintenance of PI. Our study suggests that sleep-related attentional bias in PI may be an important treatment target of CBT-I. However, the altered neural responses to SS in the current study may also reflect the reduction of sleep-related preoccupation and worry by the restoration of normal sleep after CBT-I.

Compared with GS, patients with PI showed higher activation in the precentral and prefrontal cortices in response to SS, and this was decreased after CBT-I. Increased activity in the precentral cortex, which correlated with anxiety, has been reported in insomnia even under resting conditions^16,17^. In insomnia patients, the precentral cortex showed increased connectivity to the sensory cortices^18,19^ and the amygdala^20^, suggesting a relationship of the precentral cortex with psychomotor hyperarousal and anxiety in insomnia. Our findings suggest that the hyperarousal related to precentral cortical activity in insomnia may be further exacerbated by SS and that CBT-I can prevent this exacerbation.

Previous studies have shown that the prefrontal cortex of PI patients may show decreased activity during resting or cognitive tasks^15,21^, but our study showed increased activity in response to SS. CBT-I may help to normalize this altered prefrontal activity. Prefrontal hyperactivation in response to threatening stimuli was reported to be normalized after CBT in phobia and social anxiety disorder^22,23^. Similarly, our findings suggest that CBT-I can normalize cognitive hyperarousal to sleep-related threatening stimuli by reducing prefrontal hyperactivity in PI.

Interestingly, the brain areas identified in the current study correspond largely to the default mode network (DMN). The DMN usually covers a network connecting the prefrontal cortex, PCC, and inferior parietal cortex^24^. The DMN is assumed to play a key role in worry, introspection, future envisioning, association building, and mental simulation^25–27^. During sleep, DMN connectivity has been reported to be reduced^28–30^. Increased activation of the DMN in response to SS may indicate that PI patients tend to worry about or imagine anxious and sleepless nights when sleep-related information is presented.

We also found that the reduction in response to SS in the insula after CBT-I correlated with decreased WASO after CBT-I. The insula is associated with anticipation of negative/disgusting stimuli. The insula of insomnia patients has been reported to show aberrant co-activation with the arousal network^31^. Any reduction in the insular response to SS caused by CBT-I may reflect the decreased negative emotion or arousal in response to SS as a result of CBT-I.

In the present study, the more the parahippocampal or middle frontal response to SS increased after CBT-I, the more insomnia-related dysfunctional belief was corrected. Interestingly, the direction of the correlation contradicts the notion that CBT-I normalizes brain hyperactivity in response to SS. Parahippocampal or middle frontal activation might be needed for the cognitive restructuring process regarding sleep-related dysfunctional beliefs. However, it remains unclear how CBT-I effects are related to insular, parahippocampal, and middle frontal activation in response to SS.

Limitations of the present study include the absence of non-treated PI patients. Brain imaging changes after CBT-I may be attributable to placebo rather than to true therapeutic effects. Additionally, because the control group was not tested twice, the effects of time and/or practice on the response to SS cannot be excluded. To clarify whether the change in the brain response to SS after CBT-I was caused by CBT-I itself, it would be necessary to compare the brain changes that occurred in the patient groups receiving CBT-I with those that occurred in the patient groups not receiving CBT-I during the same interval. However, there were correlations between clinical improvements and brain changes after CBT-I; thus, it is likely that the change in brain response to SS was due to CBT-I. Additionally, the clinical improvements after the last session of CBT-I were not assessed in this study. Although brain images were acquired within 11 days of the end of CBT-I, the present study could not assess long-term changes in brain responses to SS after CBT-I. Additionally, the sample size of the current study was relatively small, as this study was exploratory in nature and was designed to determine the changes in brain activity in response to SS after CBT-I. Future studies with larger sample sizes are warranted. Also, the SS used in the present study had some limitations. A new version of the SS was used following the validation performed by our research group with small number of subjects, because there were no previous SS pictures other than one emotional insomnia-related picture set. Additionally, the emotional valence of and arousal in response to each picture were not assessed in the present study. Although we used relatively non-emotional SS, anything related to sleep would produce distress in PI patients; thus, non-sleep-related and emotional stimuli were not used in the present study. However, in the absence of non-sleep related and emotional stimuli, it remains unclear whether the between-group differences reflect the selective attention to SS or the distress of patients in response to SS. Furthermore, visual stimuli consisting of sleep-related pictures are not typically presented in a dark bedroom in any real-life situation, although they can produce sleep-related anxiety in PI. Future studies using other types of SS will be required. Finally, no objective measurements of sleep after CBT-I were performed in the present study. Objective measurements, such as PSG or actigraphy, may help to clarify whether the clinical and brain changes after CBT-I were due to subjective sleep perception or objective sleep.

Normalization of psychomotor, cognitive, and emotional hyperarousal in PI may be key components of CBT-I. The current study suggests that the effects of CBT-I were mediated by reducing hyperactivity to sleep-related information in the precentral cortex, potentially related to psychomotor arousal; the prefrontal cortex, potentially related to cognitive arousal; and the insula, potentially related to emotional arousal. Modulation of activity in these areas may be a target for new insomnia treatment modalities.

An increased response to SS was found in the precentral cortex, prefrontal cortex, PCC, and fusiform cortex of drug-free PI patients. After CBT-I, and with no psychotropic medication (including hypnotics), the heightened responses to SS in the precentral and prefrontal cortexes were decreased. The clinical improvements with CBT-I correlated with a reduction in the hyperactivation to SS after CBT-I in the precentral cortex and insula. Our findings suggest that hyperarousal under sleep-related situations in PI may be related to brain hyperactivation to SS. More importantly, our study suggests that CBT-I might reduce the hyperarousal of PI by normalizing the exaggerated brain activation that occurs in response to SS.

## Methods

Initially, 25 adults (aged 18–65 years) who met the International Classification of Sleep Disorders-2 (ICSD-2) criteria for PI were recruited from an outpatient clinic at the Department of Psychiatry, and from the Center for Sleep and Chronobiology, Seoul National University Hospital. Moreover, 23 GS were enrolled via an advertisement. Exclusion criteria were 1) a past history of serious medical or neurological illness, 2) a current medical or neurological illness, 3) axis I psychiatric disorders other than primary insomnia on DSM-IV, 4) sleep disorders other than PI (based on ICSD-2 criteria), 5) short-term insomnia (duration < 6 months), 6) shift work, 7) borderline or antisocial personality disorder, 8) pregnancy, and 9) any contraindication for MRI scans.

Our study was performed in accordance with the Declaration of Helsinki regarding the ethical principles for medical research involving human subjects. The study protocol was approved by the Institutional Review Board of Seoul National University Hospital. Following provision of a complete description of the study to the subjects, written informed consent was obtained prior to study initiation. This study was conducted from May 2014 to November 2015.

To screen out psychiatric disorders, the Structural and Clinical Interview for DSM-IV (SCID-IV) was conducted with all participants, by trained psychologists. Additionally, to screen out common sleep disorders, such as obstructive sleep apnea (i.e., apnea–hypopnea index ≥ 15), nocturnal PSG was performed. Hypnotic medication may affect brain activity. To avoid potential distortion by medication and to focus on the effects of PI and CBT-I, we conducted this study under a drug-free state. Our study did not focus on patients with long-term use of hypnotics, whose brain activity may be changed by long-term medication use. Participants were requested not to take any medication that could potentially affect sleep, such as hypnotics, sedatives, antipsychotics, antidepressants, and mood stabilizers. Those who were taking these medications could participate in the study only after a 5-day drug washout period.

Of 25 PI patients, six were excluded before the initiation of CBT-I, for the following reasons: brain lesions on MRI (*n* = 2), obstructive sleep apnea on nocturnal polysomnography (PSG; *n* = 1), inability to discontinue hypnotics (*n* = 2), and withdrawal of consent during screening (*n* = 1). Of the 19 PI patients who started CBT-I, 5 withdrew from the study. Of these five patients, two refused to undergo MRI after CBT-I, and three did not complete the 5-session CBT-I. Of 23 GS, five were also excluded during screening, for obstructive sleep apnea on PSG (*n* = 2) or withdrawal of consent during screening (*n* = 3). Finally, 14 patients with PI (4 males, 10 females; mean age = 49.0 ± 12.3 years) and 18 GS (4 males, 14 females; mean age = 42.7 ± 12.3 years) were included in the analysis. There was no significance difference in clinical or demographic characteristics between the included and excluded participants. Among the 14 PI patients, 4 patients were taking zolpidem at the time of recruitment. No patient was taking other psychotropic medications. Those taking zolpidem participated in the study after a washout period ranging from 7 to 30 days. All medication tapering processes were monitored by a psychiatrist.

For the clinical assessment, both groups completed questionnaires including the PSQI, DBAS-16, and BDI. The PSQI is a self-reported questionnaire measuring overall sleep quality and includes, but is not confined to, insomnia^32^. Scores ≥ 8.5 on the Korean version of the PSQI indicate poor sleep^33^. The DBAS-16 is a self-reported questionnaire regarding sleep-related dysfunctional cognition, such as unrealistic expectations, faulty beliefs, and excessive worry regarding sleep^34^. The BDI was administered to measure depressive symptoms^35^. BDI score excluding sleep items was used for controlling depressive symptoms.

Five sessions of individual CBT-I were delivered face-to-face by two certified psychologists. Any medication potentially affecting sleep was prohibited during the entire study procedure. An eight-session CBT-I protocol^36^ was modified to be suitable for five-session CBT-I. Although the eight-session protocol includes more repetitions of sleep restriction, the main content of the protocol was unchanged. The primary treatment outcome of CBT is generally the SE according to the sleep diary^36^, which was calculated by dividing TST by TIB. Each patient was asked to go to bed only when sleepy, to get out of bed whenever they were unable to fall asleep, to wake up at the same time every morning, and to limit naps. Participants were also asked to restrict their sleep time according to their individually prescribed time-in-bed (TIB) window. Initially, the TIB window was 30 minutes more than TST and the minimal sleep window was set to 4 hours, which was relatively strict^37^. In the following session, the TIB window was titrated based on the SE of the previous week. If the SE in the sleep diary were under 85%, the prescribed TIB was decreased by 15 min. When the SE was over 90%, TIB was increased by 15 min. The prescribed TIB window at the beginning of CBT-I was 6.05 ± 0.92 h (5–7.5 h), and the prescribed TIB window at the fourth session was 6.33 ± 0.88 hours (5–7.67 h). Cognitive interventions to address dysfunctional thoughts and beliefs disturbing sleep and education regarding sleep hygiene were provided. To track changes in sleep over time, participants kept a sleep diary that included actual TIB, SL, TST, WASO, and SE. The sleep diary was collected for at least 7 days to provide baseline data before CBT-I was initiated. We continued to collect sleep diaries for each CBT-I session. Finally, patients with PI kept a sleep diary for 6 weeks (the baseline week and the five sessions of CBT-I). The baseline sleep diary before CBT-I was completed 7.07 ± 4.43 days before treatment. The sleep diary maintained between the fourth session and the last session was collected at the last session and used for the measures after treatment. The severity of insomnia was also assessed with the ISI^38^ at baseline and before and after CBT-I.

A 3-T whole-body Siemens scanner (TrioTim Syngo) with a 12-channel birdcage head coil was used for functional image acquisition with an interleaved T2*-weighted echo-planar imaging (EPI) gradient echo sequence (TR/TE/flip angle = 3,000 ms/30 ms/90°, slice thickness = 3.0 mm, in-plane resolution = 3.4 × 3.4 mm, 38 slices, field of view = 220 mm, matrix size = 64 × 64). The experiment was conducted in an fMRI room using an 8-inch screen and the stimuli were presented via DMDX software. For each participant, in total, 174 functional image volumes were acquired. After the fMRI experiment, an anatomical image was acquired using a T1-weighted, 3D gradient echo pulse sequenced with magnetization-prepared rapid gradient-echo (TR/TE/TI/flip angle = 1,670 ms/1.89 ms/900 ms/9°, slice thickness = 1.0 mm, in-plane resolution = 1 × 1 mm, field of view = 250 mm, matrix size = 256 × 256). The total duration of the experiment was ~40 min. fMRI was conducted during the daytime (between 11 AM and 3 PM) for all participants.

The fMRI experiment used a block design consisting of sleep and neutral tasks that were randomly intermixed across the run. In total, 28 sleep-related and neutral stimuli consisting of pictures taken by the current researchers and selected from the Internet were used as SS and neutral stimuli (NS; Supplementary Fig. 2). Validation of SS pictures was performed by showing sleep-related pictures to 25 outpatients with insomnia. Only pictures that more than 80% of insomnia patients indicated sleep-related were included as SS. The SS were designed to not contain or provoke any specific emotions in the general population. The NS, used as control stimuli, also consisted of pictures that did not provoke any specific emotions, but they were not related to sleep. No emotional or facial expressions were included in either the SS or NS. Both types of stimuli were matched for size and brightness.

In the stimulus epoch of each task, four SS or NS were presented for 12 s, according to the condition. Within the epoch, each stimulus was presented for 3 s. After finishing a stimulus epoch, a dot followed for 2 s and then a response stimulus was presented for 2 s. To ensure design optimization, inter-task intervals were jittered between 12 and 20 s. Each task was followed by a plus sign for 1 s to announce the beginning of the task. During presentation of the response stimuli, participants were asked to judge whether or not the stimuli were sleep-related; if they were sleep-related, they were instructed to press the right button with their right thumb; otherwise, they pressed the left button with their left thumb. The correct response rate to the entire stimulus set among all participants was 81.3%. There were no between-group differences in the correct response rate for NS (t = 1.056, p = 0.301) or SS (t = 0.059, p = 0.954). The entire run consisted of 28 SS and 28 NS, and the duration of the task was ~10 min.

fMRI data were analyzed using SPM12 software (Wellcome Department of Cognitive Neurology, London, UK). The following preprocessing steps were applied to individual participant data before statistical analysis. The first six volumes were discarded from the analysis to eliminate any effect of non-equilibrium. To correct for between-scan rigid body motion, the images were realigned to the first image in the time series. After realignment, the images were co-registered to the T1-weighted anatomical image, and normalized to Montreal Neurological Institute (MNI) space using a transformation matrix derived from the T1 anatomical image segmentation. After normalization, the images were smoothed spatially with an 8-mm full width at half-maximum (FWHM) isotropic Gaussian kernel to compensate for residual between-subject variability after spatial normalization, and to increase statistical sensitivity. The resulting fMRI time series was high pass-filtered with a cut-off time of 128 s to remove low-frequency drift. For baseline group comparisons, a voxel-based general linear model (GLM) was applied at the single-subject level to estimate parameters associated with the conditions of interest (SS, NS) in comparison with baseline, along with six motion parameters (as covariates of no interest). In detail, one regressor was used to model the SS and another was used to model the NS. The response sign and instructional cue were also modeled to reduce the residuals. Then, the design matrix was convolved temporally with a canonical hemodynamic response function (HRF) to ensure a better fit. A group-level analysis was conducted using a flexible factorial design for modeling the interaction between the group and stimulus (i.e., [SS – NS]_INS_ vs. [SS – NS]_GS_). To estimate BOLD signal changes after CBT-I, the serial fMRI data were modeled as two different sessions at the single-subject level. The follow-up MRI was performed within 11 days from the last CBT-I session. A contrast image was acquired (pair-wise contrast between before and after CBT-I; i.e., [SS – NS]_Before_ vs. [SS – NS]_After_). For the group-level analysis, the resulting contrast images were submitted to a one-sample t-test and regression analysis to characterize the linear relationship between BOLD signal changes and changes in clinical indices (e.g., DBAS, ISI, PSQI, SE, SL, TST, WASO) between before and after CBT-I. The BDI score was used as a covariate to control for the influence of depression.

To further characterize BOLD signals, subsequent region-of-interest (ROI) analyses were conducted by SPM-driven ROI analysis^39–41^. ROI masks were defined from the contrast of the baseline analysis between groups. ROIs included all significant voxels within each cluster. Signals were calculated by averaging β-values within a ROI for each subject, and then examined statistically using paired-samples *t*-tests. The ROI analysis procedure was performed using Marsbar (SPM toolbox). The threshold of statistical significance was set at a voxel-wise uncorrected p < 0.001 with a spatial extent of 20 voxels for all analyses. The more conservative thresholds used for motor and somatosensory tasks may tend to increase Type II errors in the analysis of complex cognitive and affective tasks^42^. The current study was an exploratory investigation of cognitive and affective brain responses. To achieve a desirable balance between Type I and II error rates, this threshold was set based on a prior report^37^. The locations of the peak z-values were reported using AAL anatomy (SPM toolbox). Visualization of fMRI results was performed using mricrogl.

For between-group comparisons, independent *t*-tests were used for continuous variables; Fisher’s exact test was used for categorical variables. For comparisons of continuous variables before and after CBT-I, paired *t*-tests were also used. A partial correlation analysis, controlling for age and gender, was performed to assess the relationship between clinical variables and the BOLD signals of the predefined ROIs. Analyses were performed using SPSS for Windows software (ver. 18.0; SPSS Inc., Chicago, IL, USA). All analyses were two-tailed. A p value < 0.05 was considered to indicate statistical significance.

## Electronic supplementary material

Supplementary information
