# Supplementary material for: Exploration of changes in the brain response to sleep-related pictures after cognitive–behavioral therapy for psychophysiological insomnia
Source: Sci Rep. 2017 Oct 2;7:12528. doi: 10.1038/s41598-017-13065-0 (PMC5624934; doi:10.1038/s41598-017-13065-0)

TITLE PAGE

Word count: 4,469 (text), 210 (abstract), 3 tables, and 4 figures

Supplementary information: 1 table & 3 figures

**Exploration of changes in the brain response to sleep-related pictures after cognitive–behavioral therapy for psychophysiological insomnia**

Seog Ju Kim^1^, Yu Jin Lee^2*^, Nambeom Kim^3^ Soohyun Kim^2^, Jae-Won Choi^2^, Juhyun Park^4^, Ah Reum Gwak^2^, Chang-Ki Kang^5^, Seung-Gul Kang^6^, Do-Un Jeong^2^

*^1^Department of Psychiatry, Sungkyunkwan University College of Medicine, Samsung Medical Center, Seoul, Republic of Korea*

*^2^Department of Psychiatry and Center for Sleep and Chronobiology, Seoul National University College of Medicine and Hospital, Seoul, Republic of Korea*

*^3^Neuroscience Research Institute, Gachon University, Incheon, Republic of Korea*

*^4^Department of Psychology, University at Buffalo, New York, USA*

*^5^Department of Radiological Science, Gachon University, Incheon, Republic of Korea*

*^6^Department of Psychiatry, Gil Medical Center, School of Medicine, Gachon University, Incheon, Korea*

The authors declare that they have no conflict of interest including relevant financial interests, activities, relationships, and affiliations.

^*^Corresponding author:

Yu Jin Lee, MD, PhD

Department of Psychiatry and Center for Sleep and Chronobiology

Seoul National University College of Medicine

103 Daehak-ro, Jongno-gu, Seoul 110-799, Republic of Korea

Tel.: +82-2-2072-2456, Fax: +82-2-744-7241

E-mail: leeyj1203@gmail.com, [ewpsyche@snu.ac.kr](mailto:ewpsyche@snu.ac.kr)

Supplementary Table 1. Correlation efficient (r) between BOLD response reduction after CBT-I to SS and clinical improvement of PI after CBT-I

|  | | Right precentral | | Left precentral | | Left prefrontal | | Left fusiform | | Right  PCC | | Left  PCC | |  |
| --- | --- | --- | --- | --- | --- | --- | --- | --- | --- | --- | --- | --- | --- | --- |
| Questionnaires | |  | |  | |  | |  | |  | |  | |  |
| ISI | | -0.025 | | 0.064 | | -0.282 | | -0.135 | | 0.189 | | 0.258 | |  |
| DABS | | -0.067 | | 0.174 | | 0.097 | | 0.229 | | 0.125 | | -0.069 | |  |
|  | |  | |  | |  | |  | |  | |  | |  |
| Sleep diary | |  | |  | |  | |  | |  | |  | |  |
| SL (min) | | -0.018 | | -0.022 | | 0.347 | | -0.011 | | 0.134 | | 0.059 | |  |
| TST (hr) | | -0.111 | | -0.292 | | 0.098 | | 0.165 | | -0.159 | | -0.251 | |  |
| WASO (min) | | 0.418 | | 0.628* | | 0.157 | | 0.078 | | 0.429 | | 0.409 | |  |
| SE (%) | | -0.393 | | -0.572* | | -0.182 | | 0.000 | | -0.429 | | -0.413 | |  |
| * *p* < 0.01 |  | |  | |  |  |  | |  | |  | |  | |

Note: The ROIs were defined by the areas where there were significant differences between PI patients and GS in BOLD response to SS.

Abbreviations: ISI: insomnia severity index, DBAS: Dysfunctional Beliefs and Attitudes about Sleep, SL: sleep latency, TST: total sleep time, SE: sleep efficiency, SL: sleep latency, WASO: wake after sleep onset, ROI: region-of-interest, BOLD: blood oxygen level-dependent, PI: psychophysiological insomnia, GS: good sleepers

Supplementary Figure 1. Correlation between BOLD response to SS and sleep difficulties


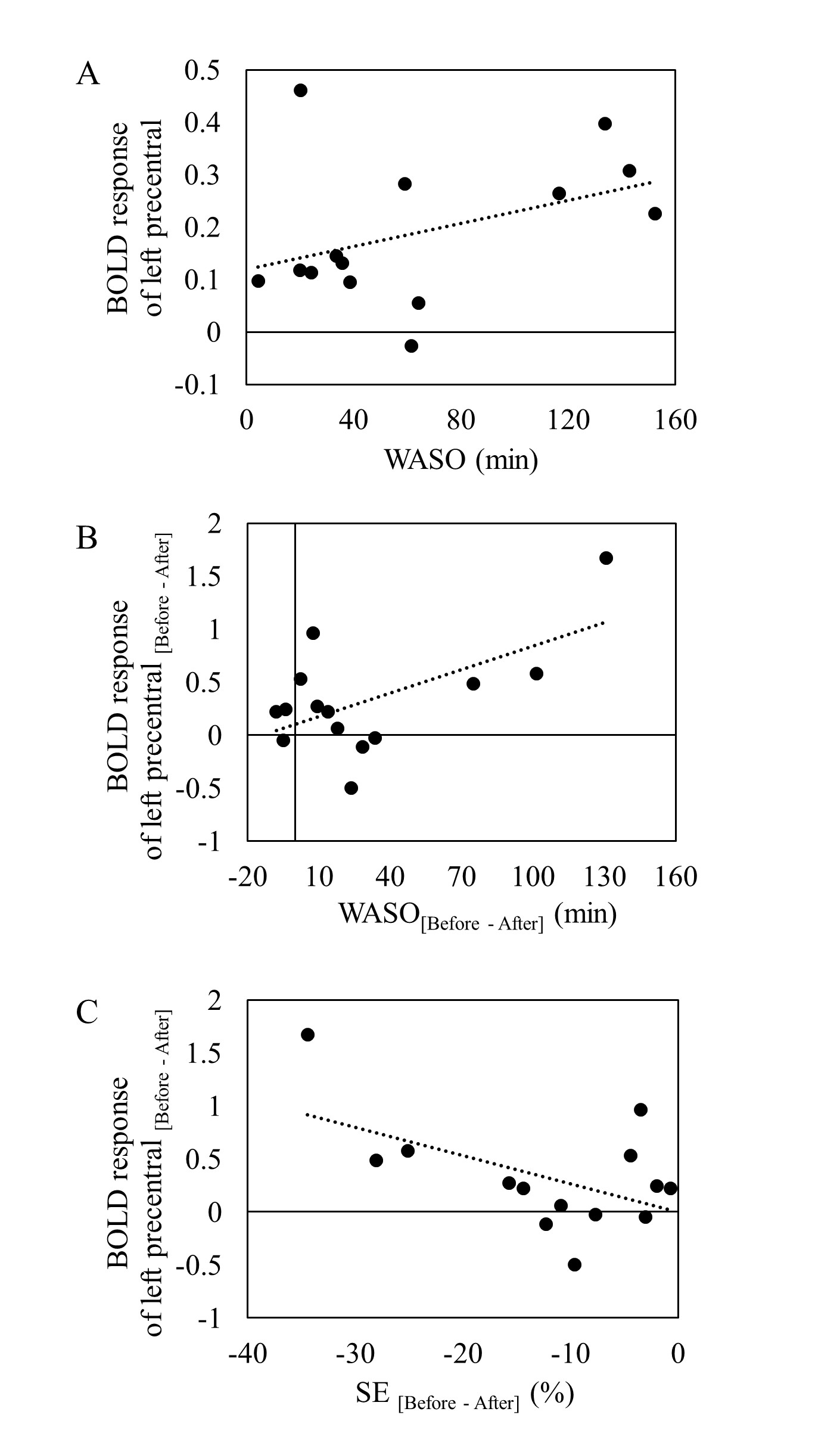


Note:

1. Correlation between BOLD response to SS in the left precentral cortex and WASO in sleep diary before CBT-I
2. Correlation between reduction in BOLD response to SS after CBT in the left precentral cortex and decrease of WASO after CBT-I
3. Correlation between reduction in BOLD response to SS after CBT in the left precentral cortex and increase of SE change CBT-I

Note: The ROI of left precentral cortex was defined by the areas where there was a significant difference between PI patients and GS in BOLD response to SS.

Abbreviations: BOLD: blood oxygen level-dependent, WASO: wake after sleep onset, SS: sleep-related stimuli, CBT-I: cognitive behavioral therapy for insomnia, ROI: region-of-interest, PI: psychophysiological insomnia, GS: good sleepers

Supplementary Figure 2. Examples of sleep-related and neutral stimuli


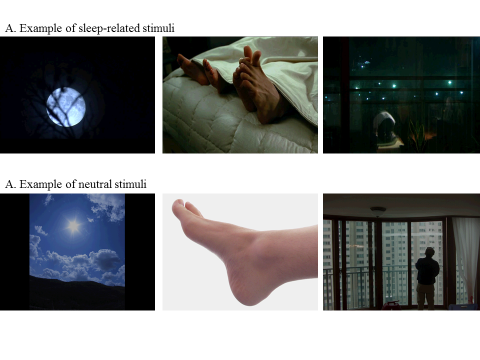

Supplement: Supplementary file 1 — Supplementary information [file 41598_2017_13065_MOESM1_ESM.doc]
